# Loop-mediated isothermal DNA amplification for asymptomatic malaria detection in challenging field settings: Technical performance and pilot implementation in the Peruvian Amazon

**DOI:** 10.1371/journal.pone.0185742

**Published:** 2017-10-05

**Authors:** Elisa Serra-Casas, Paulo Manrique, Xavier C. Ding, Gabriel Carrasco-Escobar, Freddy Alava, Anthony Gave, Hugo Rodriguez, Juan Contreras-Mancilla, Angel Rosas-Aguirre, Niko Speybroeck, Iveth J. González, Anna Rosanas-Urgell, Dionicia Gamboa

**Affiliations:** 1 Department of Biomedical Sciences, Institute of Tropical Medicine, Antwerp, Belgium; 2 Laboratorios de Investigación y Desarrollo (LID), Facultad de Ciencias y Filosofía, Universidad Peruana Cayetano Heredia, Lima, Peru; 3 FIND, Geneva, Switzerland; 4 Dirección Regional de Salud (DIRESA), Loreto, Peru; 5 Instituto de Medicina Tropical “Alexander von Humboldt”, Universidad Peruana Cayetano Heredia, Lima, Peru; 6 Research Institute of Health and Society (IRSS), Université Catholique de Louvain, Brussels, Belgium; 7 Departamento de Ciencias Celulares y Moleculares, Facultad de Ciencias y Filosofía, Universidad Peruana Cayetano Heredia, Lima, Peru; Ehime Daigaku, JAPAN

## Abstract

**Background:**

Loop-mediated isothermal DNA amplification (LAMP) methodology offers an opportunity for point-of-care (POC) molecular detection of asymptomatic malaria infections. However, there is still little evidence on the feasibility of implementing this technique for population screenings in isolated field settings.

**Methods:**

Overall, we recruited 1167 individuals from terrestrial (‘road’) and hydric (‘riverine’) communities of the Peruvian Amazon for a cross-sectional survey to detect asymptomatic malaria infections. The technical performance of LAMP was evaluated in a subgroup of 503 samples, using real-time Polymerase Chain Reaction (qPCR) as reference standard. The operational feasibility of introducing LAMP testing in the mobile screening campaigns was assessed based on field-suitability parameters, along with a pilot POC-LAMP assay in a riverine community without laboratory infrastructure.

**Results:**

LAMP had a sensitivity of 91.8% (87.7–94.9) and specificity of 91.9% (87.8–95.0), and the overall accuracy was significantly better among samples collected during road screenings than riverine communities (p≤0.004). LAMP-based diagnostic strategy was successfully implemented within the field-team logistics and the POC-LAMP pilot in the riverine community allowed for a reduction in the turnaround time for case management, from 12–24 hours to less than 5 hours. Specimens with haemolytic appearance were regularly observed in riverine screenings and could help explaining the hindered performance/interpretation of the LAMP reaction in these communities.

**Conclusions:**

LAMP-based molecular malaria diagnosis can be deployed outside of reference laboratories, providing similar performance as qPCR. However, scale-up in remote field settings such as riverine communities needs to consider a number of logistical challenges (*e*.*g*. environmental conditions, labour-intensiveness in large population screenings) that can influence its optimal implementation.

## Introduction

The accurate detection of asymptomatic and low-density *Plasmodium* infections is one of the major challenges in malaria elimination strategies [1,2]. To date, numerous studies have revealed that the currently available field-applicable tools for malaria diagnostics, *i*.*e*. light microscopy and antigen-based rapid diagnostic tests (RDTs), fail to detect a substantial part of parasite carriers, especially in areas of low to moderate endemicity [3–6]. These undetected infections act as an important reservoir for continued transmission [7] and remain as one of the primary underlying causes of unsuccessful control interventions [8]. In order to tackle this challenge, highly-sensitive diagnostic tools need to be put into the hands of pre-elimination field screening teams. This can only be achieved if truly-portable and robust methodologies allowing for detection of very low parasite densities are set-up with the aim of being suitable to national malaria control campaigns.

Molecular methods such as the polymerase chain reaction (PCR) are highly sensitive but remain too complex for field implementation due to the need for sophisticated laboratory conditions, advanced training and relatively long time to results [9]. Recent studies have shown that the loop-mediated isothermal DNA amplification (LAMP) method can deliver a similar diagnostic accuracy as PCR, without requiring complex equipment either for sample processing or for results interpretation [10]. With LAMP, DNA amplification can be performed at a constant stable temperature and the outcome of the reaction can be easily assessed by visualization of turbidity or fluorescence [11]. Different LAMP-based approaches have been successfully set-up for the detection of either *Plasmodium falciparum* (Pf-LAMP) or other human *Plasmodium* species (Pan-LAMP) [12–15]. The Loopamp MALARIA Pan/Pf detection kit (Eiken Chemical Co., Tokyo, Japan) contains strips of reaction tubes with ready-made vacuum-dried reagents, and is able to detect down to 1 parasite/μl of blood [16]. This product has been already evaluated for the detection of *Plasmodium* infections both in returned travelers [13,17] and in malaria endemic settings of different transmission intensities [18–22], displaying sensitivity and specificity ranging between 83.3–100% and 84.9–99.7% respectively, compared to PCR. However, in spite of the promising characteristics and performance of this LAMP-based tool, no study has hitherto assessed these kits at the same proximity to the point-of-care as for example the RDTs (*i*.*e*. all assays were conducted in nearby peripheral/local laboratories or within health facilities equipped with electricity supply). One of the main reasons behind is that the full LAMP assay includes a DNA extraction procedure involving sample heating and centrifugation, as well as several micro-pipetting steps, hence calling for some basic laboratory infrastructure [16].

The present study describes a LAMP implementation assessment that took place in the Peruvian Amazon region, a malaria endemic region with specially challenging logistics and epidemiological profile. First, the main *Plasmodium* species in the area is *P*. *vivax* (80% of cases) [23]. This species is characterized by very low parasite densities in blood, so it is often difficult to detect by microscopic observation [4,24]. Second, the transmission intensity in the region is low to moderate, with a significant prevalence of asymptomatic infections [25]. Third, the most commonly used malaria RDTs (based on the detection of the Histidine-Rich Protein 2; HRP2) are not recommended for species-specific diagnostic in the Amazon because of the high proportion of *P*. *falciparum* parasites presenting *pfhrp2* gene deletion [26,27]. Finally, transmission in these areas occurs mainly in hard-to-reach settings, *i*.*e*. isolated hydric communities (several-hours-distance by boat from the nearest health centre) [28] with a highly mobile population (occupational-related). The current national malaria control intervention in the area is based on Focal Screening and Treatment (FSAT) strategy, *i*.*e*. consecutive active case detection screenings in response to an unusual increase of reported cases in target communities [29]. In each screening, all community households are visited, all family members are diagnosed by light microscopy, and all individuals with a positive result receive antimalarial treatment regardless of the presence of symptoms. However, in spite of the labour-intensiveness of this strategy, it is still difficult to efficiently target and eliminate the remaining parasite reservoirs mainly due to the low sensitivity of the diagnostic tool being used in these campaigns [23].

Our main objective was to evaluate a malaria diagnostic strategy based on the use of LAMP Pan/Pf kits to detect asymptomatic infections, in the context of the screening campaigns conducted by field-mobile teams (“brigadas”) in the Peruvian Amazon. Firstly, we analysed the performance of LAMP as a tool for detecting asymptomatic infections in this epidemiological context, compared to high-sensitive molecular methods (*i*.*e*. real-time PCR as reference standard) and also to the currently used diagnostic tool for screening campaigns (*i*.*e*. microscopy). Secondly, we assessed the operational feasibility of introducing this molecular tool in the routine procedures of the mobile field teams, including riverine screenings and an on-site evaluation at a small community only accessible by boat.

## Materials and methods

### Study site

The study was conducted in Maynas province, Loreto department, Northern Peruvian Amazon. The annual average temperature is around 27°C, with an average annual rainfall of 4.000 mm and a relative humidity above 80% [30]. Malaria transmission in the area is perennial, with a peak between February and July [31] and *Anopheles darlingi* is the main vector [32,33]. *P*. *vivax* and *P*. *falciparum* infections occur at a ratio of 4/1 [23].

**Fig 1:**
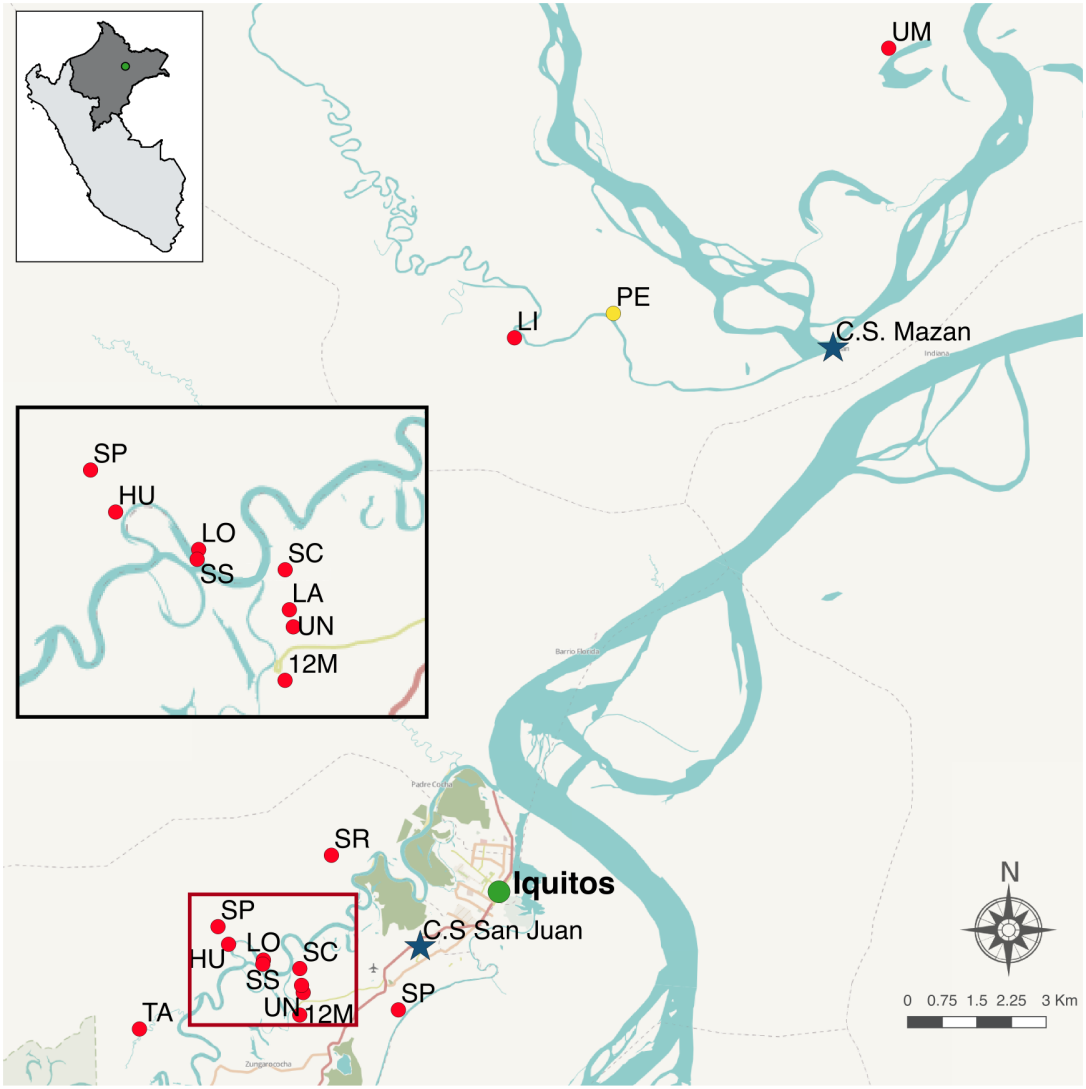
Map of the study region. Indicates the Health Centres of San Juan and Mazán (blue stars), the 13 communities included in the survey (red dots) and the pilot community for the point-of-care assay (yellow dot).

The recruitment of survey volunteers was based in two areas (Fig 1 and S1 Table). San Juan is a peri-urban area (South of Iquitos city); this screening included 11 communities scattered on both sides of the main road, 5 km around San Juan Health Centre (main health facility in the area, equipped with laboratory facilities and 24-hour electricity supply). The second one was Mazán, a riverine area (North of Iquitos city); the capital of the district, Mazán village, lies 35 km down the river from Iquitos (45 min. by speedboat) and has a Peripheral Health Centre, with a very basic laboratory facility as well as power supply restricted to morning time-slot, *i*.*e*. 9–13 a.m; this screening included 2 communities only reachable by waterway transportation (between 3–5 hours upstream by motorized boat from Mazán village). In order to simplify the terminology in the next sections, from now on San Juan will be referred as ‘road’ area and Mazán as ‘riverine’ area.

### Study design and logistics

Overall, 1167 individuals >3 years old were recruited for a prospective cross-sectional survey aimed at evaluating the use of LAMP malaria testing for active case detection among asymptomatic population, after approval by the Regional Direction of Health (Ministry of Health) in the Peruvian Amazon region. A structured questionnaire was used to determine the presence of signs and symptoms during or prior to the interview. The exclusion criteria were fever (>37.5°C) or history of fever during the past 7 days, any antimalarial intake within the last 4 weeks, and presence of at least two of the following signs of acute illness in the past 24 hours: headache, sweating, vomiting, dizziness, chills, nausea, abdominal pain, and fatigue. Field mobile teams (“brigadas”) were integrated by interviewers and nurses in charge of the data entry and sample collection, as well as microscopists and laboratory technicians dealing with the diagnostic tasks. The collection of samples was conducted during different screening campaigns between April and August 2015 (41 recruitment days in total). During the visits, study participants were diagnosed by both light microscopy and LAMP, and when any of the two tests resulted positive they were referred to the nearest health post where clinical staff proceeded according to national guidelines for antimalarial treatment. In addition, dried blood spots on filter paper were collected for further qPCR validation analysis in a subgroup of samples.

The mobile field teams’ logistics presented some differences between the two sites. During the screenings in the ‘road’ area (San Juan), the field staff moved around in pairs by “moto-car” (only for one community there was the need for a short boat trip to reach the households). Once the screening visits were completed, samples were right away transported to the Health Centre laboratory of San Juan to proceed with both the microscopy and LAMP diagnostics. For the ‘riverine’ (Mazán) campaigns the mobile teams were integrated by 9–12 members who travelled to the communities with 3 motorized boats and settled there for a few days (staying overnight in semi-closed huts and/or tents). During the screenings, the field staff moved around with the boats to reach to the scattered households (most of them surrounded by water) and, by the time samples were sequentially delivered at the community ‘base-camp’, microscopists proceeded with the blood-slide diagnostic by using mirror-illuminated microscopes (no electricity available). LAMP blood samples were transported by boat to the Mazán Health Centre laboratory twice a day. Those specimens collected during the morning screenings were delivered by early afternoon, and LAMP assays were conducted during late afternoon the same day (power supply obtained through the Health Centre’s back-up generator); those samples collected during the afternoon were stored overnight at room temperature and transported early morning to Mazán village to be processed immediately after reception.

### Parasite detection

#### Microscopy

Thick and thin films from finger-prick blood were prepared by field workers during the survey. After Giemsa staining [34], the slides were examined by a microscopist (at least 100 fields). After completion of study enrolment, two senior certified microscopists reviewed all positive slides and 10% of the negative samples (at least 200 fields). In the event of discrepancies, the smears were reread by a third expert microscopist. The result of the quality control (QC) confirmed an error rate below 5%, as recommended by WHO guidelines [35].

#### LAMP

**DNA extraction for LAMP (‘Boil and Spin’ method)**: Sixty μL of finger-prick blood were collected with a plastic capillary tube (Dropstir, Medical Precision Plastics, USA) and dispensed into a pre-aliquoted microtube containing 60 μL of LAMP lysis buffer (400mM NaCl, 40 mM Tris pH 6.5, and 0.4% SDS). Tubes were flicked to ensure mixing and stored at ambient temperature until processed. Once the samples reached the laboratory, tubes were placed in a heat-block at 95°C for 5 minutes and afterwards centrifuged at 10.000g during 3 minutes; 30 μl of the supernatant were transferred to an elution tube containing 345 μL of molecular-grade water (2/25 dilution).

**LAMP assay**: The Loopamp MALARIA Pan/Pf detection kits (Eiken Chemical Company, Japan) consist of plastic reaction tubes containing ready-made dried-down reagents for amplification of Pan *Plasmodium spp*. and *P*. *falciparum* mitochondrial target genes DNA. After blood sample processing, 30 μL of DNA elution were added to the Pan-LAMP reaction tubes, and reagents were reconstituted by mixing and inversion. Samples were incubated for 40 minutes at 65°C, followed by 5 minutes at 80°C to stop the amplification. Reaction tubes were then immediately read under a UV-lamp (D63B-Accubanker, USA). A successful amplification produces magnesium orthophosphate, a by-product of the reaction that can be detected by fluorescence (using calcein as indicator). Therefore, the LAMP assay was considered valid if fluorescence was absent in the negative control and present in the positive control. All individuals positive for Pan-LAMP were then retested using Pf-LAMP specific kits, so that the final interpretation of the positive assays allowed to differentiate between two results: (i) infected by non-*P*. *falciparum* parasites, and (ii) *P*. *falciparum* or mixed-spp. infection. In case of ambiguous fluorescence visualization, the result was recorded as indeterminate (and patients were managed according to the result of their microscopy blood smear sample). The complete standard operating procedures for the DNA extraction methods and LAMP assay are available online [16].

#### PCR

A subgroup of 503 samples including all LAMP-positive (n = 255) plus a random selection of negatives (n = 248) was analysed by two different real-time PCR methods detecting *Plasmodium*-genus and *Plasmodium*-spp infections.

**DNA extraction for PCR**: Blood spots collected on filter paper (Whatman^®^ 903 Protein Saver Snap-apart Card) were air-dried, packaged in sealable bags containing desiccant and transported to the laboratories in Iquitos and Lima for PCR analysis. DNA was extracted with the E.Z.N.A. Blood DNA Mini Kit (OMEGA Bioteck, Inc., Doraville, GA), following the manufacturer’s instructions with slight modifications. Briefly, a circle 12mm in diameter (~60–75 μl of blood) was punched out of the filter paper and incubated in TEN buffer (0.01M Tris-HCl, 0.001M EDTA, 0.1M NaCl, 0.1% SDS), followed by OB Protease buffer incubation. After addition of BL buffer and ethanol, all volume was added to the HiBind^®^ DNA Mini Column, washed 3 times and eluted in 50 μl of Elution Buffer.

**Plasmodium genus-specific real-time PCR**: Samples were screened for parasite DNA with a recently set-up real-time PCR assay targeting the *Plasmodium* genus-specific mitochondrial gene PgMt19 [36,37]. Briefly, each 20 μl reaction mix contained 5 μl of extracted DNA; the reaction was initially subjected to a hot start of 95°C for 15 minutes and then followed by 50 cycles of amplification (95°C for 15 seconds, 55° for 30 seconds and 68°C for 30 seconds). Assays were run on a CFX Connect Real-Time PCR Detection System (BIO-RAD, Hercules, CA), collecting data on the SYBR green channel. All runs included negative and positive controls. The assay was considered positive for all those samples with a Cq value <40 and melting within the expected range.

**Plasmodium species-specific real-time PCR**: A species-specific real-time PCR targeting the 18S rRNA genes of *P*. *falciparum*, *P*. *vivax*, *P*. *ovale* and *P*. *malariae* was run for all 503 selected samples, according to a previously published method [38] with slight adaptations. Briefly, 5 μl of extracted DNA were added to a 25 μl final PCR mix containing 12.5 μl PerfeCta SYBR Green FastMix 2x buffer (Quantabio, Beverly, MA) and 300 μM of each primer. The reaction started with an initial denaturation at 95°C for 2 minutes, followed by 45 cycles of amplification for 20 seconds at 95°C, 20 seconds at 52°C, and 30 seconds at 68°C. Subsequently a melt program was started consisting of 3 minutes at 68°C, and a stepwise temperature increase of 0.5°C/second until 85°C. Species were identified by melting temperature (Tm) curve analysis, based on values determined from 18S plasmid controls. The assay was performed by using a CFX Connect Real-Time PCR Detection System (BIO-RAD, Hercules, CA). All runs included negative and positive controls. PCR-positive samples were quantified by using two standard curves prepared with serial dilutions of *P*. *vivax* and *P*. *falciparum* controls of known parasite density (same filter paper DNA extraction protocol as the study samples). The assay was considered positive for those samples with a Cq value <39 and melting curve within the expected range.

Two External Quality Control assessments with blinded samples, including negative and positive external controls submitted by WHO (n = 10; from 18 to 10,000 parasites/μl) and by FIND (n = 8; from 1 to 20 parasites/μl), confirmed correct performance for the two real-time PCR techniques employed in this study.

### Operational feasibility assessment

During the cross-sectional study, we evaluated the feasibility of integrating a LAMP-based diagnostic methodology within the logistics of field-teams in charge of screening both terrestrial and hydric Amazonian communities. The following parameters were assessed: (a) infrastructure and material requirements; (b) complexity of the procedure, *i*.*e*. training needs, difficulty of the protocol; (c) throughput capacity; (d) complexity of results interpretation, *i*.*e*. inter-observer variability assessment by computing the number of differing results among two blinded readers; and (e) time-to-result. In addition, at the end of the survey, the laboratory staff in charge of conducting the LAMP assays were submitted to individual interviews.

**Point-of-care (POC) LAMP testing**: The applicability of LAMP in the context of very isolated field settings without any lab infrastructure was assessed by performing a separate pilot assay in a small riverine community named 1° de Enero (Fig 1). This 121-people community is only reachable by waterway transport, has no electricity supply nor cell phone coverage, and is at a 2-hour distance of the nearest Health Centre by motorized boat.

### Data analysis

Data were double entered into FIND Vision*Form* study databases and analysed using STATA 14 (StataCorp, 2015. Stata Statistical Software: Release 14. College Station, TX) and R v.3.2.2 (R Development Core Ream, R Foundation for Statistical Computing, Australia). Infection prevalences were derived with 95% Confidence Interval (CI), and differences between groups were compared using Fisher’s exact test. Sensitivity, specificity and accuracy of the methods under evaluation were based on the comparison with a ‘composite reference standard’ (*i*.*e*. combination of two real-time PCR assay results; a sample was considered positive when at least one of the two PCRs gave positive result). Differences among test performance variables in different groups were compared using Fisher’s exact test. Cohen’s Kappa coefficient was calculated in order to assess the agreement among different diagnostic methods, accounting for random effect. Negative binomial regression was used for significance testing of continuous skewed data (*i*.*e*. parasite density) [39]. P-values < 0.05 were considered statistically significant.

### Ethics statement

The protocol was approved by the Ethical committee board at Universidad Peruana Cayetano Heredia (SIDISI code 63991). All participants provided informed consent and participants under 18 years old provided in addition the assent form.

## Results

### Characteristics of the study population

The demographic characteristics of the 1167 voluntary asymptomatic participants are shown in Table 1. The median age of the participants was 22 [Interquartile Range = 3–39] and 18% (207) of the samples were collected in riverine communities.

**Table 1:** Baseline characteristics of the study population.

	N	%
**Age**:		
3–5 years	141	12.1
6–18 years	392	33.6
>18 years	634	54.3
**Gender**:		
Female	636	54.5
Male	531	45.5
**Area**:		
River	207	17.7
Road	960	82.3

### Prevalence of asymptomatic malaria by microscopy and LAMP

The prevalence of malaria among asymptomatic population was 4.9% (95% CI 3.72–6.28) and 21.9% (95% CI 19.59–24.44) according to field microscopy and LAMP, respectively (Table 2). *Plasmodium falciparum* was detected in 10.5% (6/57) of the positive microscopic samples (either as mono-infection or mixed species), whereas *P*. *vivax* accounted for the majority of infections. Out of the Pan-LAMP positive samples, 13.7% (35/255) were also positive by the Pf-LAMP assay (indicating *P*. *falciparum* or mixed infection). Five (0.4%) LAMP assays presented an indeterminate result, 4 of them occurring in riverine LAMP screenings.

**Table 2:** Detection of malaria infections by microscopy and LAMP, and species identification.

	MICROSCOPY [n = 1167]			LAMP [n = 1162^a^]	
	*N*	*% (95% CI)*		*N*	*% (95% CI)*
**Malaria prevalence**:	57	4.9% (3.72–6.28)		255	21.9% (19.59–24.44)
**Relative proportion of species**:					
*P*. *vivax*	51	89.5% (78.48–96.04)	Non-*falciparum*	220	86.3% (81.43–90.25)
*P*. *falciparum*	5	8.8% (2.91–19.30)	*P*. *falciparum* or *mixed infection*	35	13.7% (9.75–18.57)
*Mixed infection*	1	1.7% (0.04–9.39)			

LAMP, loop-mediated isothermal amplification; CI, confidence interval

^a^5 LAMP results not available (indeterminate reading)

### Performance of LAMP and microscopy compared to a reference qPCR standard

A subgroup of 503 samples (all LAMP positives and a random selection of negatives) was further assessed with the aim to evaluate the performance of LAMP compared to a reference qPCR standard, as well as the accuracy of the current diagnostic tool in use (*i*.*e*. microscopy). Overall, 256/503 (50.9%; 95%CI = 46.4–55.3) resulted positive at least by one of the two qPCR assays, *i*.*e*. composite reference standard (Table 3). Details on the concordance between the two independent qPCR methods are provided in S2 Table.

**Table 3:** Detection of malaria infections by real-time PCR, species identification and parasite densities.

	qPCR results (N = 503)	
**Positive samples by qPCR composite reference standard** [N; %]	256	50.9%
**Positive samples by Mitochondrial-qPCR** [N; %]	254	50.5%
**Positive samples by 18S-qPCR** [N; %]	239	47.5%
**Relative proportion of species**^a^ [N; % (95% CI)]		
*P*. *vivax*	205	85.8% (80.69–89.94)
*P*. *falciparum*	33	13.8% (9.70–18.84)
*Mixed infection*	1	0.4% (0.01–2.31)
**Mean parasite densities**^a^ [geometric mean (95% CI)]		
*Overall*	9.97	(7.46–13.31)
*P*. *vivax*	10.65	(7.77–14.59)
*P*. *falciparum*	6.59	(3.09–14.08)
*Mixed infection*	NA	NA

CI, Confidence Interval; NA, Not available (estimation not feasible due to multiple species)

^a^Results from 18S-qPCR

LAMP had a sensitivity of 91.8% (87.7–94.9) and specificity of 91.9% (87.8–95.0) when compared to qPCR, while microscopy had a sensitivity of 20.3% (15.6–25.8) and specificity of 98.0% (95.3–99.3) compared to the qPCR reference standard (Table 4). Overall, the agreement of qPCR with LAMP was much higher than with microscopy (0.873 and 0.180 kappa coefficient, respectively).

**Table 4:** Performance of LAMP and microscopy, compared to the qPCR reference standard.

		qPCR reference standard^a^		Sensitivity (95% CI)	Specificity (95% CI)	Accuracy (95% CI)	Kappa coef. (95% CI)
		NEG.	POS.				
		N = 247	N = 256				
**LAMP**	**NEG**.	227 (91.9%)	21 (8.2%)	91.8% (87.7–94.9)	91.9% (87.8–95.0)	91.85% (89.1–94.1)	0.837 (0.79–0.88)
	N = 248						
	**POS**.	20 (8.1%)	235 (91.8%)				
	N = 255						
**Field MICROSCOPY**	**NEG**.	242 (98.0%)	204 (79.7%)	20.3% (15.6–25.8)	98.0% (95.3–99.3)	58.45% (54.0–62.8)	0.180 (0.13–0.23)
	N = 446						
	**POS**.	5 (2.0%)	52 (20.3%)				
	N = 57						

^a^ Composite reference standard, based on the combination of results of 2 quantitative PCRs (qPCRs), *i*.*e*. 18S and Mitochondrial

Among the 20 LAMP-positive qPCR-negative samples, 1 of them was also diagnosed as positive by microscopy (field screening and QC confirmation; 4,780 parasites/μl). On the other hand, among the 21 samples that were LAMP-negative but qPCR positive, 3 of them were diagnosed as positive by both field and QC microscopy readings (densities: 5,441 parasites/μl, 3,721 parasites/μl and 78 parasites/μl).

#### Performance according to parasite density

**Fig 2:**
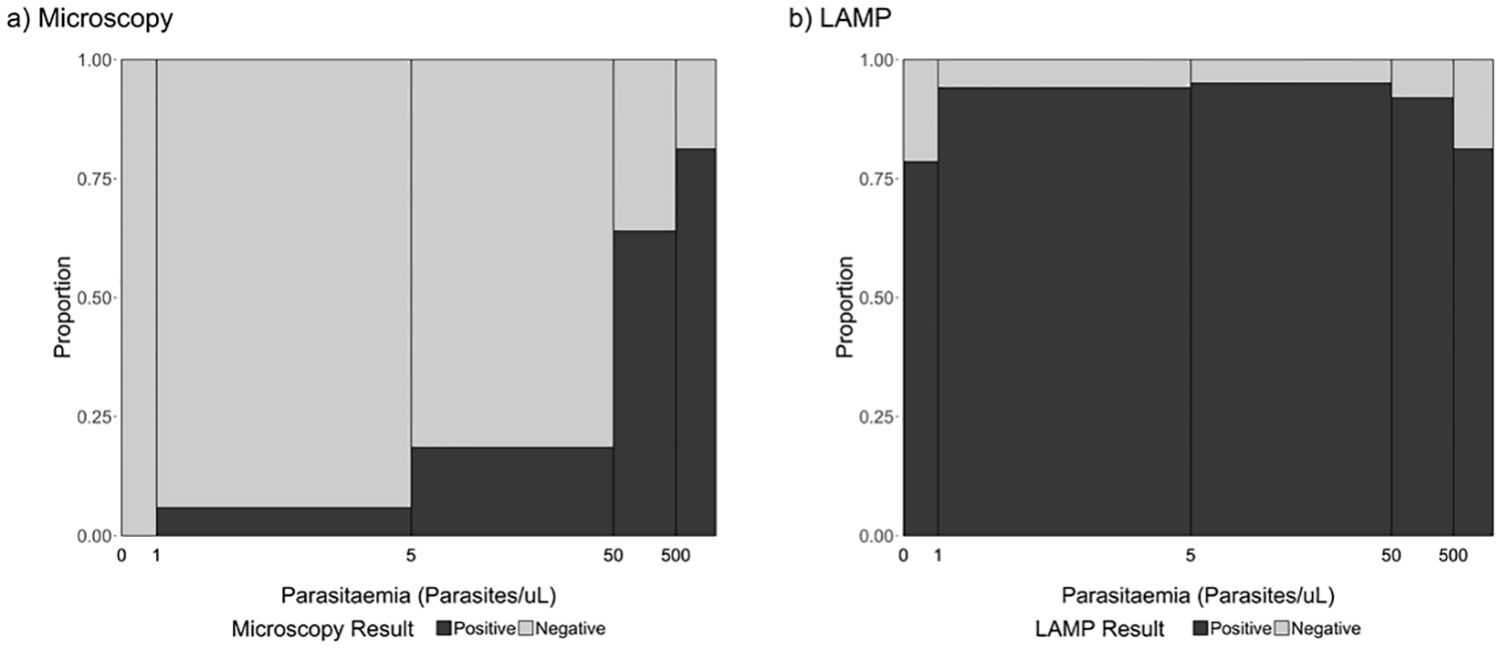
Proportion of qPCR-confirmed infections that were detected by microscopy (a) and LAMP (b), according to parasite density. The bar widths are proportional to the number of cases within each parasite density category. N = 238 (samples with parasite density data available [18S - qPCR]).

The failure to detect infections was found to be associated with the parasite density of the sample. As shown in Fig 2, the chances of microscopy to detect an infection decreased dramatically for samples presenting less than 50 parasites/μl (as per qPCR) in comparison to higher densities (p<0.001). For LAMP determinations, we observed a moderate–not significant–decrease in the sensitivity when parasitemias were below 1 parasite/μl (78.6%) but also above 500 parasites/μl (81.2%), compared to the ranges in between (92.0–95.1%).

#### Performance according to study area

A stratified analysis of the performance results obtained in road and riverine screenings was conducted. Overall, the proportion of River samples included in the qPCR sub-analysis (19.3%) was equivalent to the general study recruitment (17.7%). Riverine screenings presented a slightly higher prevalence of qPCR-positive samples (56.7% vs. 49.5%), and also a wider presence of *P*. *falciparum* infections than road samples (23.4% vs. 12.0%), although none of these differences was statistically significant (Table 5). The parasite densities of *P*. *vivax* positive samples were also comparable in the two study sites, whereas *P*. *falciparum* infections presented higher parasite densities among riverine samples compared to those detected in road samples (Coeff = 1.64; 95% CI 0.24–3.05; p = 0.021). The performance of the two evaluated methodologies was significantly different between the two study areas. The accuracy of both microscopy and LAMP techniques was better among the samples collected during road screenings than the samples from riverine communities (p≤0.004).

**Table 5:** Performance of LAMP and microscopy in the two study areas, compared to the qPCR reference standard.

		ROAD	RIVER	p-value^a^
		*N (%)*	*N (%)*	
Total samples (N = 503)		406 (80.7%)	97 (19.3%)	**-**
Positive samples^b^ (N = 256)		201 (49.5%)	55 (56.7%)	0.215
Percentage of *P*. *falciparum*/Mixed^c^ (N = 239)		22 (12.0%)	11 (23.4%)	0.092
		*% (95% CI)*	*% (95% CI)*	
LAMP	Sensitivity	95.0% (91.0–97.6)	80.0% (67.0–89.6)	0.001
	Specificity	94.1% (90.0–96.9)	80.9% (65.9–91.4)	0.009
	Accuracy	94.6% (91.9–96.6)	80.4% (71.1–87.8)	<0.001
Field MICROSCOPY	Sensitivity	22.9% (17.3–29.3)	10.9% (4.1–22.2)	0.059
	Specificity	99.5% (97.3–100.0)	90.5% (77.4–97.3)	0.003
	Accuracy	61.6% (56.7–66.3)	45.4% (35.2–55.8)	0.004

CI, Confidence Interval

^a^ Fisher′s exact test

^b^ According to the qPCR Reference Standard

^c^ According to the result of the species-specific qPCR (18S)

### *Plasmodium* species identification by LAMP

The *Plasmodium* species identification of LAMP-positive samples presented high agreement with the species-specific qPCR results (94.15%; 225/239). Particularly, among the 205 samples that were diagnosed as *P*. *vivax* infection by qPCR, 8 (3.9%) of them were identified as *P*. *falciparum*/mixed by LAMP. Among the 34 samples that were diagnosed as *P*. *falciparum* or mixed infection by qPCR, 6 (17.6%) of them were identified as non-*falciparum* by LAMP. All *P*. *falciparum*/mixed samples that Pf-LAMP failed to detect were low parasite density infections (below 4 parasites/μl).

The *Plasmodium* species distribution among samples that went undetected by LAMP was not different than the species distribution in the general study population (p > 0.05).

### Operational feasibility of LAMP assays in the field

The field-suitability of the malaria LAMP-based diagnostic strategy was evaluated at different levels.

#### Infrastructure, equipment and material requirements

A stable power supply source (during daytime), and a flat working surface for equipment and sample processing were the primary infrastructure requirements. All materials were stored in a cool and dry cupboard (*i*.*e*. LAMP kits are stable between 4° - 30°C, and include desiccant inside each sealed pouch), hence continuous cold chain maintenance was not necessary. Monitoring of the environmental temperature and humidity was conducted in the laboratory of Mazán—where the climatic conditions are more extreme—showing an oscillation between 26.9°C-28.3°C and 69%-80% humidity. The essential equipment for the execution of the assay was a microcentrifuge, a thermoblock (with 1.5mL and 0.2mL tube adaptors) and a UV-lamp. Since the commercial kit already contained vacuum-dried components for LAMP amplification reaction, only house-made lysis buffer and molecular-grade distilled water were required as additional reagents for the DNA extraction procedure.

#### Complexity of the assay

A theorico-practical training of 2 days was sufficient to teach two local laboratory technicians to perform the full LAMP procedure, including DNA extraction, sample amplification and results interpretation. The protocol involves pipetting of micro-volumes (*e*.*g*. 30 μl) and implementation of basic preventive measures to avoid cross-contamination among samples. Both technicians had limited previous experience on the use of molecular techniques but were able to carry out autonomously all LAMP assays of the study, without the need for additional assistance nor close supervision.

According to the interviews’ feedback, the appraisal from the laboratory staff was generally positive; LAMP was perceived as a rather simple tool to provide molecular diagnostic results for field teams within a short turnaround time, compared to PCR conducted in national reference or research laboratories. In terms of practicability, the two interviewees coincided that the main bottleneck of the assay was the DNA extraction labour-intensiveness when large amounts of samples are accumulated.

#### Throughput capacity

Each run of LAMP assays was performed in batches of maximum 46 samples, plus a positive and a negative kit control (determined by the capacity of the heat-block). The average duration of the DNA extraction procedure was approximately 1 hour, whereas the LAMP amplification assay and subsequent result reading took normally less than 1 hour. A full testing round (*i*.*e*. extraction, Pan assay and Pf assay, results annotation) had a total duration of 2.5–3.5 hours, depending on the batch size, as well as the number of positives that required species confirmatory run. It is also important to highlight that real ‘hands-on-time’ (*i*.*e*. involving active bench work) was shorter than the total duration estimation since the protocol includes incubation periods. The maximum number of LAMP testing rounds that was experimented during the study was 3 full assays in one day, allowing for the analysis of 130 samples by one technician.

#### Results interpretation

To assess the inter-observer variability, a total of 305 samples were submitted to a secondary fluorescence reading by the laboratory technician that had not been conducting the assay. Among all the double-readings, 8 samples (2.62%) presented a discordant result: in all events the reported results differed between “negative” in first reading and “indeterminate” in the second, thus being rated as minor discrepancy errors. A third reading of the discordant samples was conducted by a trained lab supervisor, and all 8 samples were finally scored as "negative”.

During the study, the presence of DNA extraction products with haemolytic appearance was detected regularly among the samples coming from riverine screenings, sometimes reaching up to 50% of the sample collection batch. When these samples were processed by the ‘Boil and Spin’ method, the resulting supernatant appeared as intensive red (instead of clear yellow), conferring a reddish background to the final LAMP reaction that difficulted the clear visualization of fluorescence as well as the naked-eye turbidity assessment.

#### Time-to-result

The results of the LAMP assays conducted during the riverine screenings could be ready within the next 24 hours after sampling, whereas LAMP results from road screenings were available within less than 12 hours post-sampling.

#### ‘POC-LAMP testing’ in the community

**Fig 3:**
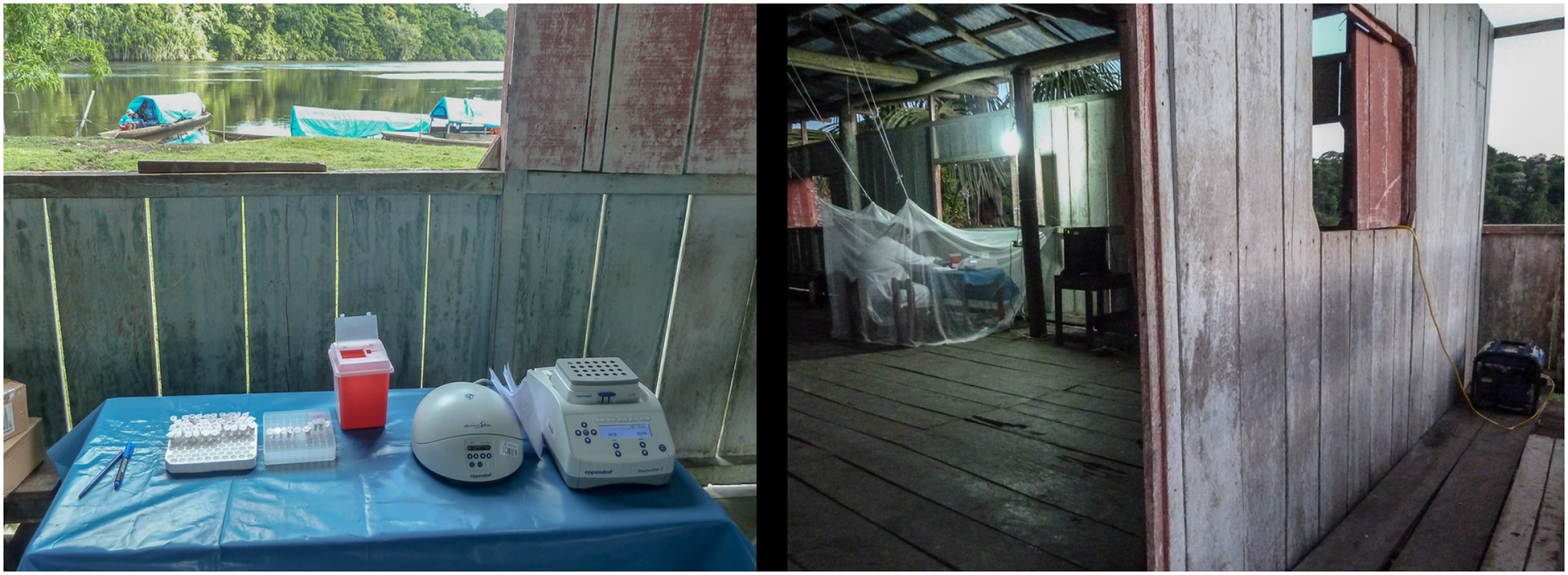
Laboratory set-up for ‘POC-LAMP testing’ in the community.

In order to be able to conduct the LAMP assay outside a laboratory facility, an inventory of equipment and materials was packed and subsequently transported to the target community by a traditional motorized boat. Once on site, the ‘pop-up’ laboratory was set-up in the elevated non-floodable roof-covered space normally serving as the public shed where villagers gather for events in this type of communities. The essential equipment requirements were: a fuel portable power generator; a current stabilizer; the laboratory devices (thermoblock, centrifuge, UV-lamp); a hanging lamp for lighting after sunset; and long electric cables or extensions. Other gear to optimize the set-up were a king-size mosquito net and a big piece of fabric/plastic to cover the ground surface and block the entrance of insects from underneath the raised floor. The full set-up of the testing space took approximately 1 hour (Fig 3).

The team of field workers collected 45 samples from nearby households and these were processed just upon reception. The full LAMP assay took a bit less than 3 hours, and results were immediately transferred to the field personnel in order to proceed according to the corresponding national guidelines. None of the samples collected during this pilot presented haemolytic appearance after DNA extraction. Temperature and humidity were periodically monitored during the 24 hours stay in the community, reaching a maximum temperature of 32.6°C and maximum humidity of 94%.

## Discussion

### Performance of microscopy

Efforts to control and eliminate malaria require reliable tools for detecting very low parasite densities among asymptomatic patients in order to promptly identify and treat any parasite carriers. This study shows that a considerable amount of circulating parasites are not detected by the diagnostic method currently employed during the malaria control campaigns of the Peruvian Amazon, revealing a much a higher malaria prevalence in the area compared to estimates of the National Programme [40]. In particular, LAMP detected 4.5 times more *Plasmodium* positive samples than microscopy in the general population, and the comparative analysis indicated that microscopy only provides acceptable performance for the detection of infections presenting densities above 50 parasites/μl. Likewise, another study conducted in Colombia also reported a dramatically increased detection of asymptomatic malaria infections by LAMP as compared to light microscopy [19]. These observations call for the need to revise the current malaria surveillance system relying exclusively on microscopy, since inefficient diagnostic strategies can undermine control interventions’ success, specially in low-transmission settings such as Latin American endemic regions [41].

### Performance of LAMP

Real-time PCR was used as the reference standard to confirm the presence or absence of parasite DNA in a subset of specimens that had been previously screened by LAMP. In accordance with previous studies reporting performance of the Pan/Pf LAMP detection kits (Eiken) [13,17–19], the technique showed good sensitivity and specificity results (>90%) compared to qPCR. The proportion of LAMP false negative results was found to be low (8.2%) specially if considering that the definition of positive sample in this analysis was very stringent (*i*.*e*. composite standard based on two independent high-sensitive qPCR assays). LAMP also detected 19 positive samples that were not confirmed using qPCR and microscopy. This finding could be explained by the comparable detection threshold between LAMP and the reference standard, and in fact similar observations have also been reported in previous LAMP assessments conducted in Zanzibar [20] and Uganda [18]. In terms of *Plasmodium* species identification, the overall agreement among LAMP and qPCR was high (>94%), and discrepancies were more frequently observed for samples identified as *P*. *falciparum* by qPCR. This finding is consistent with other studies [20] and with the previously reported detection limits of the Pf-LAMP and Pan-LAMP kits [16], being the former moderately higher (~1.5 times) hence explaining that some low density *P*. *falciparum* infections could be correctly detected by Pan-LAMP, but not by the succeeding Pf-LAMP reaction.

Subsequent stratified analysis of the performance results revealed several interesting observations. On the one hand, most LAMP detection failures occurred at very low (<1 p/μl) or very high (>500 p/μl) parasite densities. One possible explanation for the high-parasitaemia failures would be an inhibitory effect of high DNA concentrations on the LAMP reaction; to our knowledge this has never been reported in previous LAMP studies, so an ad hoc assessment with a larger number of hyperparasitaemic samples would be required to further investigate this observation. Secondly, LAMP diagnostic accuracy was found to be significantly better among road screenings than in riverine screenings. When comparing the parasite burden among samples of the two study areas, a significant difference was observed for *P*. *falciparum* parasite densities; however the trend indicated that riverine communities presented higher parasitemias, so this observation would fail to explain the hindered performance in this study setting. The factors underpinning the haemolytic appearance of riverine samples may also have influenced the diagnostic accuracy by interfering on the performance of the LAMP reaction and/or the visualization of fluorescence. In line with this hypothesis, the proportion of indeterminate LAMP results was found to be significantly higher among the riverine screenings (1.9%) than among road screenings (0.1%), p = 0.004. The reasoning behind the hindered result visualization for ‘reddish’ LAMP specimens has not been addressed so far; however it could be speculated that this is related to the quenching activity of haemoglobin against fluorophores such as calcein [42]. The occurrence of haemolytic appearance could be associated to the operational particularities of the specimen collection in the riverine area, where sample boxes were carried around during hours by field staff (usually in a backpack, under continuous movement), transported by long boat trips and kept for longer storage periods at room temperature. On the other hand, road screening samples were transported by land in small portable fridges and accounting for shorter delays to get to the laboratory. In this regard, previous LAMP validations with clinical field samples had not raised a similar observation on haemolytic appearance of DNA extraction products [18–22], probably because the operational conditions for sample collection were not as challenging as in the remote riverine field setting selected for this assessment.

Also in agreement with the operational causality hypothesis, during the POC-LAMP assay conducted in the community there was no single specimen presenting haemolytic appearance, probably because this riverine screening did not involve continuous sample shaking combined with assay delays. Although the sample size of this pilot experience is too limited to draw definitive conclusions and the qPCR verification procedures were not identical to the general validation study, a posterior analysis of the subset of POC-LAMP samples suggested that assays conducted in the same community could even provide a slightly improved performance, compared to the general riverine screenings comprising laboratory-based LAMP testing (overall accuracy 84.4% *vs*. 80.0%, respectively).

### LAMP as a tool for national malaria screenings

One of the main goals of this study was to assess the feasibility of integrating LAMP as a surveillance tool for national malaria screening campaigns. Furthermore, this pilot LAMP implementation included for the first time riverine communities, which represent a high proportion of population at-risk in the Amazon region. Overall the use of a LAMP-based diagnostic method proved to be compatible with the routine procedures of the control programme. It was demonstrated that technicians without strong experience in molecular tools could perform wide-scale LAMP testing without difficulty, in modest laboratory spaces equipped only with a few simple appliances. The total hands-on-time of the LAMP assay was comparable to that of field microscopy, and involved a much easier result interpretation, *i*.*e*. no high specialization required; the interreader agreement on reaction fluorescence was high (>97%) and no major discordances (negative *vs*. positive) were reported.

However, several considerations should be taken into account for the deployment of large-scale LAMP screenings in remote field settings: (i) the procedure requires minimum staff training, both for the specific method to collect the blood sample with a plastic capillary tube (field nurses) and for the LAMP assay protocol (laboratory technicians); (ii) the distance and accessibility to the LAMP testing site is determining, not only for the time-to-result but also because long sample storage and/or transport could affect the specimen quality; and (iii) high-throughput performance depends on the DNA extraction capacity (bottle-neck if high number of samples, and only one technician and/or equipment set available). Further simplification of the sample processing protocol was noted as a main priority by the staff participating in the study screenings. Centrifugation-free commercial methods for DNA extraction of LAMP samples have been already designed; however, in spite of the good clinical sensitivity results obtained for some of these platforms [14,43], their performance as high throughput systems for low-density infections screening in the field has not proved to be successful [44], or they are not even compatible with testing of large number of samples [14].

Another limitation to consider for the large-scale deployment of LAMP is the current cost of the kit [45]. However this could be balanced by the quick turnaround time that this strategy can offer for the molecular detection of unaverted low-density infections in remote settings, compared to the delay and costs required for sending samples to a PCR reference lab, where highly-skilled personnel needs to be available.

### ‘POC-LAMP’ testing at community level

A possible strategy to prevent sample deterioration and to reduce the overall turnaround time would be conducting the assays in the same community where the screening takes place, instead of referring them to the nearest laboratory-based facility. In this study we have proved that accurate LAMP testing can also be performed without the need of a stable laboratory infrastructure, and that a mobile team can transport and arrange the necessary set-up to allow the assay running either very near or at the same ‘point-of-care’ level. Importantly this proof-of-concept demonstration took place during a population screening in a remote community only accessible by boat and without electricity supply, but could be applicable to an extensive network of extremely isolated Amazonian hydric communities where there is currently no chance to be part of any ‘screen & treat’ strategy based on molecular malaria diagnostic. The ‘POC-LAMP’ approach conferred therefore several advantages, *i*.*e*. time-to-result < 5 hours (compared to 24h required for referred testing in the Peripheral Health Centre lab of Mazán) and reduced risk of sample deterioration; however this strategy also involves a number of additional challenges to deal with. Firstly, the need to move and install all equipment and gear for every setting which is logistically more demanding. Secondly, the environmental conditions can be more extreme when work is conducted outside the lab; during the pilot assay, temperature reached above 30°C and humidity went above 80%, so additional measures such as kit transportation in portable fridges (with coolers) and storage into plastic containers with desiccant could be recommended. Also the use of measures to avoid insect annoyance and work interference was indispensable. Finally, the operational feasibility of this strategy can be highly-influenced by the climatic conditions, specially if implemented in riverine areas, where rainfall regimes determine for example the water level and thus the boat accessibility to isolated communities, *e*.*g*. limiting the disembarkment of staff and materials.

## Conclusions

This study has demonstrated that LAMP-based diagnostic can provide accurate detection of asymptomatic malaria parasite carriers in an epidemiologically challenging setting such as the Amazon basin, including isolated communities without laboratory infrastructure.

The LAMP malaria kit is a promising tool intended for use in remote field settings, even near or at the same POC level, which could render increased efficiency to current malaria control interventions. However, there are a number of logistical challenges that can hinder its optimal implementation. Among others, the deterioration of collected blood samples should be avoided to prevent ambiguous fluorescence and turbidity interpretation, unless an alternative method allowing for simple, robust and affordable result reading (*e*.*g*. colorimetry) is made available.

## Supporting information

S1 Table List of communities included in the study. (DOC)

S2 Table Agreement among the results of the two real-time PCR methods. (DOC)

S1 Dataset Study dataset. (XLSX)
