# Supplementary material for: Loop-mediated isothermal DNA amplification for asymptomatic malaria detection in challenging field settings: Technical performance and pilot implementation in the Peruvian Amazon
Source: PLoS One. 2017 Oct 5;12(10):e0185742. doi: 10.1371/journal.pone.0185742 (PMC5628891; doi:10.1371/journal.pone.0185742)
Supplement: S1 Table — (DOC) [file pone.0185742.s001.doc]

**S1 Table**. **List of communities included in the study.**

| **SCREENING** | **COMMUNITY** | **AREA** | **POPULATION** | **POSITION (UTM Zone 18)** |
| --- | --- | --- | --- | --- |
| ‘Road' screenings | LAGUNA AZUL (LA) | San Juan / Iquitos | 375 | 684534 9580685 |
|  | TARAPOTO (TA) | San Juan / Iquitos | 210 | 677071 9579252 |
|  | SANTA CLARA de NANAY (SC) | San Juan / Iquitos | 1836 | 684435 9581497 |
|  | HUATURI (HU) | San Juan / Iquitos | 130 | 677180 9582511 |
|  | 12 de MAYO (12M) | San Juan / Iquitos | 216 | 684483 9579096 |
|  | UNION (UN) | San Juan / Iquitos | 720 | 684627 9580333 |
|  | SAN PEDRO (SP) | San Juan / Iquitos | 260 | 684843 9585034 |
|  | SANTA RITA (SR) | San Juan / Iquitos | 480 | 686139 9587151 |
|  | SAN PABLO de CUYANA (SP) | San Juan / Iquitos | 115 | 680245 9583552 |
|  | LOBOYACU (LO) | San Juan / Iquitos | 87 | 678711 9585359 |
|  | SANTA SOFIA (SS) | San Juan / Iquitos | 50 | 682622 9581787 |
| ‘Riverine’ screenings | URCOMIRAÑO (UM) | Mazán | 364 | 715048 9628294 |
|  | LIBERTAD (LI) | Mazán | 347 | 696143 9613302 |
| ‘Point-of-care' screening | 1ero de ENERO (PE) | Mazán | 121 | 700016 9615105 |
