# Supplementary material for: Loop-mediated isothermal DNA amplification for asymptomatic malaria detection in challenging field settings: Technical performance and pilot implementation in the Peruvian Amazon
Source: PLoS One. 2017 Oct 5;12(10):e0185742. doi: 10.1371/journal.pone.0185742 (PMC5628891; doi:10.1371/journal.pone.0185742)
Supplement: S2 Table — (DOC) [file pone.0185742.s002.doc]

**S2 Table. Agreement among the results of the two real-time PCR methods.**

|  |  | **Mitochondrial qPCR** | |  | |
| --- | --- | --- | --- | --- | --- |
|  |  | **NEGATIVE** | **POSITIVE** | ***Total:*** | |
| **18S qPCR** | **NEGATIVE** | 247 | 17 | *264* |  |
|  | **POSITIVE** | 2 | 237 | *239* |  |
|  | ***Total:*** | *249* | *254* | **503** |  |
|  |  |  |  |  |  |
| **Kappa Coef.** | 0.924; CI 95%= 0.891 - 0.958 | | |  |  |
